# The role of dietary fibre in pig production, with a particular emphasis on reproduction

**DOI:** 10.1186/s40104-018-0270-0

**Published:** 2018-08-06

**Authors:** Selene Jarrett, Cheryl J. Ashworth

**Affiliations:** The Roslin Institute and R(D)SVS, University of Edinburgh, Scotland, EH25 9RG UK

**Keywords:** Fibre, Pig, Pregnancy, Production, Reproduction

## Abstract

Fibres from a variety of sources are a common constituent of pig feeds. They provide a means to utilise locally-produced plant materials which are often a by-product of the food or drink industry. The value of a high fibre diet in terms of producing satiety has long been recognised. However the addition of fibre can reduce feed intake, which is clearly detrimental during stages of the production cycle when nutrient needs are high, for example in growing piglets and during lactation. More recently, fibre has been found to promote novel benefits to pig production systems, particularly given the reduction in antimicrobial use world-wide, concern for the welfare of animals fed a restricted diet and the need to ensure that such systems are more environmentally friendly. For example, inclusion of dietary fibre can alter the gut microbiota in ways that could reduce the need for antibiotics, while controlled addition of certain fibre types may reduce nitrogen losses into the environment and so reduce the environmental cost of pig production. Of particular potential value is the opportunity to use crude fibre concentrates as ‘functional’ feed additives to improve young pig growth and welfare. Perhaps the greatest opportunity for the use of high fibre diets is to improve the reproductive efficiency of pigs. Increased dietary fibre before mating improves oocyte maturation, prenatal survival and litter size; providing a consumer-acceptable means of increasing the amount of saleable meat produced per sow. The mechanisms responsible for these beneficial effects remain to be elucidated. However, changes in plasma and follicular fluid concentrations of key hormones and metabolites, as well as effects of the hypothalamic satiety centre on gonadotrophin secretion and epigenetic effects are strong candidates.

## Background

Dietary fibre, usually defined as the indigestible portion of food derived from plants, forms a key component of many pig diets. Although not fully digested, dietary fibre can affect a wide range of physiological processes, both directly (e.g. by gut fill) and indirectly by the production of gases and physiologically active by-products following fermentation in the colon. Dietary fibre also changes the nature of the contents of the gastrointestinal tract, which in turn affects how other nutrients and chemicals are absorbed. In addition, dietary fibres can bind steroid hormones in the digestive tract, affecting entero-hepatic circulation and altering the balance between steroid excretions and circulating concentrations. A variety of fibrous feedstuffs are routinely added to pig diets, usually depending on local availability of low-cost fibre-rich ingredients which are often by-products from the food and drink industries. In temperate regions of the world, these include distillers dried grains, soybean hulls (SBH), wheat bran, sunflower meal and sugar beet pulp. With increasing global demand for livestock feed, novel co-products from tropical food industries, including copra (coconut) meal, palm kernel, rice bran, kiwi fibre and canola meal are increasingly used. It is important to note that differences in the nutritive properties of different fibre sources exist, including differences in amino- and fatty acid profiles.

The objective of this review is to bring together current information of the use of fibre-rich ingredients in contemporary pig diets and to consider the impact of such diets on pig production efficiency, welfare and the environment. The earlier sections of the review will consider the impact of feeding high-fibre diets to pigs at all stages of the production process. The primary focus of the review will be to assess the contribution of dietary fibre to improved pig reproductive performance and to consider the potential mechanisms involved. Opportunities to refine both the precise duration of feeding high fibre diets and the most effective fibre sources will be assessed.

### Sources and types of dietary fibre

Dietary fibre includes a wide range of carbohydrates known as non-starch polysaccharides including pectins, cellulose, hemicelluloses, β-glucans and fructans as well as oligosaccharides and starch that are resistant to hydrolysis in the small intestine. The physiological properties of different fibres are related primarily to their solubility, viscosity, physical structure and water-holding capacity, rather than their constituent monomers. Classically, dietary fibres have been categorised as either soluble fibres, which are fermented in the colon to produce gases and physiologically active by-products, or as insoluble fibres which are metabolically inert and provide bulk to the diet.

### The use of fibre in pig diets

World-wide, a diverse range of fibre-rich ingredients are added to pig diets. They include wheat bran, wheat middlings, oat husks, maize bran, rye bran, sugar beet pulp and fibre, corn cobs and bran, distillers grains, rapeseed, soy bean hulls, kiwi fruit and chicory. Fibrous crop by-products, forages and roots including cassava, citrus pulp, konjac flour and sweet potato are frequently used in sub-tropical and tropical countries. Although these diets do not always maximise pig performance, they provide an effective and economical use of locally grown feedstuffs and hence contribute to sustainable production. The inclusion of dietary fibres in pig diets is sometimes limited because they can have ant-nutritive properties. These include a reduction in the digestibility of dietary energy and protein [1] which may lead to inadequate amino acid, particularly threonine, absorption [2]. Although fibrous dietary constituents can have higher crude protein levels than non-fibrous constituents, on average approximately 30% of nitrogen is bound to neutral detergent fibres and therefore not available to the animal [3]. Various methods were proposed to reduce the anti-nutritive properties of dietary fibres. They include reducing particle size to improve digestibility, de-hulling or scarifying to reduce tannin content, and heat treatments to reduce heat-labile anti-nutritive factors (reviewed by Woyengo et al. [4]).

The digestibility coefficient of dietary fibre varies from 0.40 to 0.60, compared with other nutrients (protein, fat, sugars or starch) which have values above 0.80. Variability in the digestibility of dietary fibre depends on the fibre source. For example, wheat straw is poorly digested because of the presence of lignin, whereas sugar beet pulp is more digestible because of the presence of pectic substances [5].

The relative benefits and drawbacks of including fibre in pig diets depend on the stage of the production process and on the production system. For example, the impact of the inclusion of dietary fibre depends on the maturity of the animals receiving such diets. Dietary fibre may compromise the ability of growing pigs to obtain adequate energy, it appears to have little impact on energy retention in finishing pigs [6] and is often used to promote satiety in gestating sows. This reflects the greater development and larger gastro-intestinal tract, lower feed intake per kg of body weight, slower digesta transit time and higher cellulolytic activity of adult pigs compared with young pigs (reviewed by Lindberg, [7]). The use of forages rich in dietary fibre was also proposed for more extensive systems, including tropical extensive production systems where the ability to reduce urinary nitrogen emission is of great interest as a means to promote nutrient recycling [3].

### Effects of fibre on the digestive tract and intestinal health

The pig gastrointestinal tract adapts to diets high in dietary fibre, although this can take several weeks. Martinez-Puig et al. [8] estimated that the gastrointestinal tract of growing pigs takes 5 wk to adapt to a raw potato starch diet, as assessed by whole tract digestibility and faecal excretion. Therefore, the duration of consumption of a high fibre diet will alter the impact on the digestive tract. Dietary fibres are, by definition, not hydrolysed by endogenous enzymes in the small intestine and are therefore available for bacterial fermentation in the large intestine. Dietary fibre significantly modifies the microbial equilibrium in the intestine, with positive or negative impacts on animal health depending upon the source of the dietary fibre and the physiological status of the pig. For example, the addition of guar gum or cellulose to a standard pig diet increased ileal populations of Bifidobacteria and Enterobacteria populations in growing pigs [9]. More recently, it was suggested that with declining use of antimicrobial growth promoters, selective inclusion of fibres into pig diets could alter the gut microbiome and promote gut health [7]. Indeed, recent studies using the pig as a model to study the effects of human diets on gut microbiota [10] demonstrated that high-fibre diets based on wheat bran increased copy numbers of ‘beneficial’ bacteria including lactobacilli and bifidobacteria, while the low fibre diet fostered bacterial groups associated with a negative impact on gut health.

Intestinal bacteria hydrolyse dietary fibres and metabolise their constituent sugars leading to the production of ATP which is used for basal metabolism and growth of gut bacteria. The primary end products of microbial fermentation of dietary fibre are short-chain fatty acids (acetate, propionate and N-butyrate) and gases (carbon dioxide, hydrogen sulphide and methane). The relative proportions of short-chain fatty acids produced vary depending on the type of dietary fibre [3]. The short-chain fatty acids released by anaerobic bacteria following fibre fermentation contribute to the animal’s energy supply and regulate both the growth of gut epithelial cells and the composition of the gut flora. In an acidic environment, short-chain fatty acids can inhibit the growth of enteric bacterial pathogens. The energy produced from hindgut fermentation varies depending on the carbohydrate content of the diet while the contribution of fibre to a pig’s energy requirement depends on the pig’s maturity. It was estimated that fermentation products contribute 0.15 for growing-finishing pigs [11] and 0.3 for gestating sows [12]. Regardless of fibre source and the animal’s maturity, the contribution made by short-chain fatty acids produced by fermentation of dietary fibre to an animal’s total energy requirements is considerably less than from glucose following an equivalent intake of digestible starch [1].

Dietary fibre and the short chain fatty acids produced as a consequence of fibre digestion are believed to have effects on the structure and function of the intestine. In pigs these changes include increased gastrointestinal tract weight [13], villus height and crypt depth [14, 15], and goblet cell number [16] and greater capacity of the intestine for oxidative metabolism [17]. Butyrate was shown to induce differentiation and apoptosis in the small intestine, increase intestinal cell proliferation in piglets and improve the digestive and absorptive capacities of the pig small intestine [18]. A more recent study compared the effects of different fibre types on intestinal cell differentiation [19] and showed that feeding diets high in wheat straw or corn distillers dried grains with solubles to finishing pigs increased the percentage of the mucosal area containing goblet cells and altered the expression of nutrient receptors and transporters when compared to control pigs fed a corn-soybean diet.

In addition, physical properties of dietary fibre, such as the ability to absorb and hold water and the viscosity and solubility of the digesta can affect digestion, satiety and feed transit time. Dietary fibre, particularly soluble dietary fibre, can increase retention time in the stomach, causing earlier satiety due to distention of the stomach wall. However, the bulking capacity of dietary fibre reduces the transit time of feed in the small and large intestine, reducing the duration of exposure of the diet to intestinal digestive enzymes, the digestibility of other nutrients in the diet and the incidence of constipation [20]. The bacterial growth supported by fermentation induces a shift in nitrogen excretion from urine to faeces [21], which, as discussed later, is associated with reduced environmental cost of pig production.

## Effects of dietary fibre on aspects of pig production

### Behaviour and welfare

In contemporary production systems, pregnant sows are typically fed a restricted diet during gestation in order to avoid excess weight gain and associated farrowing and locomotion problems [22]. However, this is believed to result in pregnant pigs being hungry, which is a welfare concern. Furthermore, conventional diets are consumed within minutes of being offered and the sow’s feeding motivation remains high [23]. Consumption of diets containing bulky fibres increases postprandial satiety [24] as a consequence of gut-fill and delayed gastric emptying, the release of satiety-inducing gut peptides and the increased availability of short chain fatty acids in the distal gut coincident with a reduction in post-prandial glucose absorption. High fibre diets can also reduce post-prandial activity of the pig [25], including the incidence of non-feeding oral [26] and other stereotypic [27] behaviours. High fibre diets were therefore proposed as a means to promote nutritional satiety and reduce apparent feeding motivation, thereby improving the welfare of sows subjected to feed restriction during pregnancy. However, these results are equivocal, with some studies showing no effects of a high fibre diet on salivary cortisol concentrations, stereotypic behaviours [28] or feeding motivation [29]. The type of dietary fibre consumed affects the degree of satiety [27]. These last authors proposed that fibres with a slow rate of fermentation and high production of butyrate, such a tapioca starch, would be most satiating.

### Immune status

McGlone and Fullwood [30] reported that pregnant gilts receiving a diet containing 25% sugar beet pulp had more white blood cells. However, other humoral and cellular immune markers (natural killer cell cytotoxicity, neutrophil chemotaxis and chemokinesis, mitogen-induced lymphocyte proliferation and differential counts) were unaffected. Feeding diets high in crude fibre during pregnancy, but not lactation, decreased levels of C-reactive protein (a marker of inflammation) in colostrum [31]. In growing pigs, there is some evidence that pigs fed a sugar beet fibre diet have reduced faecal egg counts following an experimental challenge [32].

### Nitrogen excretion and environmental impact

Dietary fibre is a means to reduce the environmental burden associated with pig production systems. Studies in rats have shown that consumption of increased dietary fibre creates intestinal conditions that favour the transfer of urea from the blood to the large intestine [33]. As a consequence, urinary nitrogen excretion is decreased while faecal nitrogen output increased. The breakdown of protein in manure takes weeks or even months, whereas the degradation of urea to ammonia and CO_2_ takes several hours. Indeed, both Nahm [34] and Aarnink and Verstegen [35] reported that dietary fibre reduced ammonia emission. Kreuzer et al. [36] showed that feeds with high pectin and hemicellulose content, such as citrus pulp and sugar beet pulp, were the most effective dietary fibre sources to reduce nitrogen loss in manure. Using prediction equations, Toma et al. [37] indicated that adopting a high fibre diet based on sugar beet pulp prior to mating, as described in Ferguson et al. [38], would reduce the impact of such systems on the environment (air and groundwater pollution) by about 6% (namely 6.34% for greenhouse gas emissions of methane and nitrous oxide and 6.23% for nitrate losses through leaching/runoff into groundwater).

### Reproduction

The greatest benefits of feeding pigs diets high in fibre may be achieved via improved reproductive outcomes. Even modest increases in the number of piglets weaned per sow per year have considerable commercial advantages. Numerous studies assessed the impact of diets containing varying levels of dietary fibre on fertility at all stages of the porcine reproductive cycle. Most promising results seem to be obtained when high fibre diets are fed prior to mating.

#### Feeding dietary fibre prior to mating

There is a wealth of evidence showing that the diet consumed by gilts and sows before mating can have major effects on prenatal survival in the ensuing pregnancy and ultimate litter size. This is believed to be because the oocyte developing within the ovarian follicle is very sensitive to changes in maternal nutrient intake, which in turn affects circulating hormone and metabolite levels, thereby altering ovarian function [39]. Additionally, oocyte maturation is sensitive to disruptions and perturbations in maternal health and well being (reviewed in Krisher [40]). Therefore, high fibre diets fed from the beginning of the oestrous cycle could have a positive impact during this maturation process.

Analyses of the in vitro maturation (IVM) of oocytes can be used to assess their development to the critical MII stage of meiosis. This is the stage at which the oocytes have the developmental competence to be fertilised and to produce a good quality embryo. Gilts fed a high fibre diet (containing 50% USBP) from d 1 to 19 of their third post-pubertal oestrous cycle had more oocytes that reached MII following 46 h of IVM culture compared to control gilts [41]. Additionally, pre-pubertal gilts fed a high fibre lupin diet from 3 wk before puberty stimulation until d 19 of their first oestrous cycle had almost nine times more MII oocytes than gilts fed the low fibre control diet and more than five times more MII oocytes than the gilts fed the medium fibre bran diet [42]. Despite the alterations in oocyte quality, dietary fibre does not appear to affect ovulation rate [42–45].

Embryos recovered from gilts fed a high fibre diet (50% USBP) in their first oestrous cycle developed into blastocysts with more cells than embryos of control (cereal) fed pigs [45], and more embryos were recovered from the high fibre fed gilts than the control gilts [45]. Following in vitro fertilisation (IVF), blastocysts produced from oocytes collected from gilts fed a 50% USBP diet from d 1 to 19 of their third oestrous cycle had more cells than blastocysts produced from oocytes collected from control (barley-based diet) fed pigs [46]. This suggests that feeding a high fibre diet before mating has an influence on early embryo development, with blastocysts derived from both in vivo fertilisation and IVF having more cells, and therefore being better developed.

Ferguson and Leese [47] confirmed the beneficial effects of feeding dietary fibre during the oestrous cycle preceding mating on embryo survival. When gilts were fed either different amounts of a maintenance (M) diet (1.8× M or 2.6× M) or a diet high in either USBP, protein or starch prior to insemination, only high fibre fed gilts showed increased embryo survival and decreased incidence of foetuses with retarded growth on d 30 [43]. Such reproductive benefits were also observed following consumption of alternative fibre sources. Embryo survival was higher on d 27-29 after mating in gilts fed a lupin-based diet prior to insemination [41, 42]. Feeding a high fibre diet prior to mating also improved the outcome of pregnancy. More piglets were born and more were born alive when sows were fed a 20% USBP diet from d 11 of lactation until weaning on d 25 followed by a 40% USBP diet from weaning to oestrus [38].

#### Feeding dietary fibre during gestation

Several studies have investigated the effects of feeding a high fibre diet to pigs during gestation on pregnancy outcome. Renteria-Flores et al. [44] fed gilts one of four diets from 28 d before mating until 28-35 d of gestation. They used a corn-soybean (CSB) meal control diet and CSB diets containing either 30% oat bran (soluble fibre), 12% wheat straw (insoluble fibre), or 21% SBH diet (high in soluble and insoluble fibre). The number of live embryos and total embryo survival rate was lower in sows fed the diets containing any insoluble fibre compared to sows fed the control and just soluble fibre [44]. It is likely that these benefits to embryo survival could be attributed to specifically feeding the insoluble fibre prior to mating as opposed to also during early gestation. This is supported by the fact that these last authors did not observe any effect on litter size when the sows were fed from 2 d after insemination to d 109 of gestation [44].

Other studies confirmed that feeding a high fibre diet during gestation does not affect litter size. For example, supplementing a fortified sorghum-soybean meal diet with 25% beet pulp, or a CSB meal-based diet with 0.3% psyllium, 20% SBH, or 20% SBH with 13 % wheat middlings during gestation did not affect farrowing rate, litter size at birth, or the number of stillborns [30, 48, 49]. Sun et al*.* [50] fed sows a diet based on konjac flour twice a d during gestation and three times a d during lactation, and reported no difference in the total number of pigs born, the number of pigs born alive, stillborns or mummified foetuses [50].

However, few studies did report contradictory changes in litter size. Sows fed a CSB meal based feed supplemented with 40% SBH one d after weaning until d 109 of the following pregnancy had fewer pigs born compared to sows fed a non-supplemented feed [28]. Conversely, feeding sows a diet high in fermentable non-starch polysaccharides during the weaning to oestrus interval and the subsequent pregnancy over three successive parities, resulted in an increase in the total number of piglets born and the number of live born piglets [51].

The number of piglets weaned may also be affected by feeding dietary fibre during gestation. Sows fed a high fibre diet containing 13.35% ground wheat straw over three successive gestation-lactation cycles farrowed and weaned 0.51 more pigs per litter than sows fed a control CSB meal diet [52]. However, other studies contradicted this finding, with no effect of feeding konjac flour or different amounts of crude fibre during gestation on the number of pigs weaned [50, 53].

There are inconsistencies in the effects of feeding high fibre diets during gestation on litter performance. Total litter birth weight and weaning weights were 0.87 kg and 3.59 kg greater respectively, in offspring of sows fed wheat straw compared to the offspring of control sows [52]. Sows fed ad libitum a diet enriched with sugar beet pulp, oat bran and oats, after weaning their first litter until the next lactation, weaned piglets that were heavier than those of control fed sows [54]. However, pregnancy rate, weaning to oestrus interval, the number of piglets born alive or number of stillborn piglets were not affected by treatment. Conversely, sows fed psyllium from breeding until d 4 post-partum weaned lighter pigs than sows fed the control diet [48], despite no treatment effect on birth weight. Multiparous sows fed a diet based on konjac flour, throughout lactation, showed no difference in litter weight or average pig weight at birth [50].

Various studies assessed the effects of feeding pregnant sows diets differing in fibre content on the growth rate of piglets. There were no effects of diet on litter size or weight at birth, However, piglet weight gain from 1 to 5 d of age was higher in offspring from sows fed a diet containing 7% versus 3.8% crude fibre from late pregnancy (d 95) until d 5 of lactation [20]. A similar observation was made when sows were fed increasing levels of several fibre sources, including sugar beet pulp, from gestational d 25 until farrowing. Piglets from fibre fed sows grew faster than piglets from control sows [55]. Similarly, when a high fibre diet was fed to primiparous sows from the 5 wk of gestation until farrowing, piglet growth rate during the first post-natal week and weight at weaning increased [56]. However, Loisel et al. [57] reported that feeding a high fibre diet from d 106 of pregnancy until parturition did not influence piglet weight gain until d 21 of lactation. Additionally, feeding sows a konjac flour diet during pregnancy resulted in no difference in average daily gain and no difference in average pig weight or litter weight at d 21 of lactation [50].

#### Effects of feeding a fibre diet during lactation on suckling piglets

Few studies specifically assessed the effect of feeding a high fibre diet during lactation on the performance of suckling piglets because fibrous ingredients may reduce feed intake and are therefore often excluded from lactation diets. In a study reported by Renaudeau [58], a high fibre diet (20% neutral detergent fibre) fed to sows during lactation increased litter growth rate and piglet body weight at weaning, but was also associated with a greater loss of sow body weight over lactation. However, in a more recent study, it was reported that feeding high fibre diets containing sugar beet pulp or alfalfa meal from d 109 of gestation and throughout lactation had detrimental effects [59]. Specifically, the sugar beet pulp diet impaired sow feed intake and reduced piglet weight gain at peak lactation. Although milk yield was unaffected by treatment, milk protein content was reduced in sows fed alfalfa meal.

#### The effect of dietary fibre on different parities

Two studies reported that the benefits of feeding a high fibre diet differed according to parity. In one study, sows were allocated high, middle or low fibre diets for two parities from mating to 2 d after parturition [53]. In the first parity, high fibre diets increased litter size but decreased mean individual birth weight [53]. In the second parity, mean birth weights of piglets was higher in higher fibre sows, whilst total litter weight at parturition was lower and mean growth of individual piglets from 3 d to 8 wk was slower in litters from high fibre sows [53]. A similar study was carried out where gilts were allocated either a low, mid or high fibre diet from d 1-90 of gestation over two parities [60]. In the first parity, low fibre sows had more total pigs born and more pigs born alive whilst in the second parity, mid fibre sows had more total pigs born and pigs born alive [60]. Within litter, uniformity in birth weight was higher in mid and high fibre sows in the first parity only [60]. In the first parity, litter weight and average pig weights at parturition and on d 22 of lactation were greater in low and mid fibre sows, but in the second parity, high fibre sows had more live pigs and had higher litter weight at parturition [60].

Collectively, the results suggest that to improve future reproductive performance, a high fibre diet is most advantageous when fed prior to mating. The most likely reasoning is that the high fibre diet affects the very early stages of development of the oocyte and embryo, rather than factors such as ovulation and fertilisation rates.

### Possible mechanisms involved in the beneficial effects of a high fibre diet on pig reproduction

**Fig. 1:**
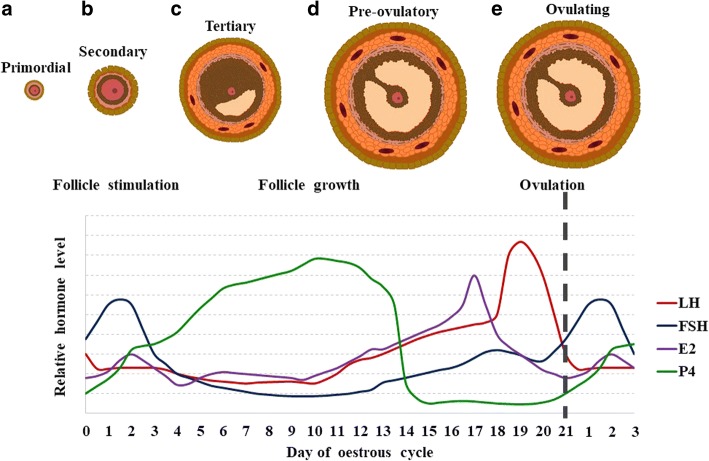
Reproductive hormone profile during the porcine oestrous cycle and the corresponding stages of folliculogenesis. Profile of luteinising hormone (LH, red), follicle stimulating hormone (FSH, blue), oestradiol (E2, purple) and progesterone (P4, green) during the oestrous cycle of the pig and the corresponding stages of folliculogenesis. The initiation of the primordial follicle (**a**) to a secondary follicle (**b**) is induced by the increase in FSH which is followed by the expansion of the secondary follicle to the tertiary (antral) follicle (**c**) and then eventually to the pre-ovulatory follicle (**d**). This is accompanied by the increase in P4 which is involved in preparing the endometrial lining, and the increase in E2 which initiates the rapid release of LH. This LH surge results in the rupture of the follicle, an event known as ovulation (**e**). The relative hormone levels show the within hormone changes during the cycle; E2 concentrations are in pg/mL whilst P4, FSH and LH are in ng/mL

The underlying mechanisms by which a high fibre diet improves fertility remain elusive. A high fibre diet alters the circulating concentrations of oestradiol and luteinising hormone (LH), reproductive hormones are important for oocyte maturation and pregnancy establishment [41]. Oestradiol is produced by the granulosa cells of growing follicles during the oestrous cycle and induces the hypothalamic-pituitary (HP) axis to release LH, therefore triggering ovulation (Fig. 1). Gilts fed high fibre diets had lower circulating oestradiol concentrations on d 17, 18 and 19 of their oestrous cycle as well as increased LH pulse frequency on d 18 and higher LH peaks [41, 45]. The decrease in circulating oestradiol reduces the negative feedback effects on the HP axis, thereby increasing LH pulse frequency.

#### Reduction in circulating oestradiol

Oestrogens in plasma can either be bound to proteins or be free, and hence biologically active at the target cells. The main active oestrogen is oestradiol-17β (E_2_), which is metabolised in the liver to oestrone, and to a lesser extent, oestriol. In the liver, oestrogens are conjugated to glucuronic acid and/or a sulphate group, making them water soluble. Excess conjugated oestrogens in the plasma are then excreted in urine or excreted into the intestines as bile. However, about 50% of the conjugated oestrogens are excreted from the hepatic system into the intestine in bile. After deconjugation in the intestinal lumen, about 80% of these oestrogens are reabsorbed via the intestinal wall and vena porta into the liver. In humans, a higher intake of dietary fibre was associated with a reduction in circulating oestradiol and greater faecal oestrogen [61].

Oestradiol has the capacity to strongly bind to various fibre types (including cholestyramin, lignin, and cellulose) and sources (including wheat bran, cereals, seeds, and legumes) [62]. Faecal excretion of both free and unconjugated oestrogens was three times higher in rats after 24 h of feeding a wheat bran based diet (11% fibre content) compared to rats fed a white wheat flour based diet (0.5% fibre content) [63]. Arts et al. [64] also studied the effect of feeding male rats a non-fibre wheat starch diet, a low-fibre wheat flour diet or a high-fibre wheat bran diet on oestrogen excretion after injection with C^14^ labelled E_2_. Wheat bran fed rats excreted approximately twice the amount of E_2_ in faeces during the first d after injection and had an increased rate of faecal excretion of E_2_ after 3 wk compared to rats fed the other two diets [64].

#### Neuropeptide Y and satiety

Another possible mechanism by which the high fibre diet may exert its effects could involve the neurotransmitter, neuropeptide Y (NPY). NPY was documented to be involved in the regulation of food intake and in the secretion of gonadotrophin releasing hormone (reviewed in McDonald, [65] and Wójcik-Gładysz and Polkowska [66]). A high fibre diet increases gut fill [67] and hence could activate the NPY system, including effects on hypothalamic gonadotrophin releasing hormone (GnRH) secretion. Although levels of NPY were not measured in high fibre fed pigs, nuclear magnetic resonance (NMR) studies comparing the metabolic profiles of plasma from pigs fed a diet either high in starch or in lipid and fibre revealed that leptin concentrations were lower in high fibre pigs [68], suggesting that leptin may regulate feed intake in these cases. The relationships between NPY, leptin and both GnRH release and gonadotrophin secretion in pigs fed a high fibre diet require further study, particularly given the lower circulating oestradiol concentrations in pigs fed a high fibre diet and the fact that NPY stimulates the release of LH and follicle stimulating hormone (FSH) in the anterior pituitary in the presence of oestrogen [69].

#### Circulating metabolites

Another potential mechanism involves changes in concentrations of metabolites in plasma and subsequently ovarian follicular fluid. For example, circulating levels of insulin-like growth factor-1 (IGF-1) and leptin were lower in high fibre fed pigs [42]. Pigs fed a high lipid, high fibre diet had lower plasma concentrations of β-hydroxybutyrate, leptin, glucose, insulin and urea in one study [68]. However, in contrast to Jégou et al. [68], Yde et al. [70] found no clear effect of dietary fibre on glucose and insulin responses overall, but found that insulin concentration was highest in sugar beet pulp fed pigs compared to potato pulp and pectin. The high fibre diet resulted in increased plasma short-chain fatty acids and non-esterified fatty acids, and a negative correlation was found between the amount of fibre in the diets and plasma creatine [70]. The study also identified biomarkers in plasma for the different fibre types; methylsulfonylmethane (DMSO_2_) for pectin, scyllo-inositol and DMSO_2_ for potato pulp, and scyllo-inositol, DMSO_2_, and betaine for sugar beet pulp [70]. This is of particular interest as betaine is a methyl donor that has been implicated in epigenetic processes.

#### Betaine, methylation and epigenetics

Alterations to the diet occurring prior to, or soon after, mating can affect gene expression patterns in oocytes and embryos which may persist throughout the pre- and post-natal life of the resultant individual, a process termed epigenetics. Oocyte maturation is a crucial period for the erasure, acquisition and maintenance of genomic imprints. These changes are mediated, at least in part, by the pattern of gene methylation. Because the methylation imprint in females is established at the time of oocyte maturation, the metabolic state of females during ovarian follicular growth can exert important effects on the methylation process, (reviewed by Sinclair et al. [71]).

Betaine is a trimethyl glycine derivative, involved in epigenetic processes, in particular deoxyribonucleic acid (DNA) and histone methylation (reviewed in Anderson et al*.* [72]). Dietary betaine, or choline oxidised into betaine, donates a methyl group to homocysteine, converting it into methionine whilst betaine itself is converted into dimethylglycine with the enzyme betaine-homocysteine methyltransferase (BHMT). Methionine is then converted into the methyl donor S-adenosylmethionine (SAM), the major methyl donor of DNA methylation [73].

Supplementation of drinking water with betaine increased betaine levels in mouse plasma, muscle and liver [74]. However, the plasma betaine levels were elevated further in mice fed both betaine and the soluble fibre polydextrose [74]. Feeding a high fibre diet was also associated with increased plasma betaine concentrations in pigs [75]. Proton NMR-based spectroscopy identified higher intensities of signals from methyl protons from betaine in hypercholesterolemic pigs fed high fibre content rye bread [76]. However, it is worth noting that betaine absorption is four to six times slower in pigs fed a high fibre diet compared to pigs fed a direct-betaine supplemented diet [75].

One study contradicts these results, whereby NMR spectroscopy-based analysis of plasma showed higher plasma betaine concentrations from sows fed a diet low in fibre compared to sows fed diets high in soluble and insoluble fibre [77]. However, the study included only six sows, where the three diets were fed in a repeated 3 × 3 cross-over design, which could potentially lead to a carry-over of dietary effects. Interestingly, the study was carried out by the same researchers that identified betaine, along with dimethyl sulfone and scyllo-inositol, as a biomarker for feeding sows sugar beet pulp [70]. The earlier plasma metabolomic study incorporated a larger number of animals; 48 gestating primiparous sows with 12 sows per treatment group, adding confidence to the initial result suggesting that betaine is a useful biomarker for feeding sugar beet pulp.

In the ovary, it is thought that betaine is transported through cumulus cells before gap junctions close during meiotic maturation, and the betaine in MII oocytes is then regulated by the signalling threshold-regulating transmembrane adapter 1 (SIT1) transporter that arises post-fertilisation [78]. There is evidence indicating that BHMT activity works in conjunction with the folate cycle in methylation processes in both oocytes and blastocysts. Mean numbers of inner cell mass (ICM) cells of mouse blastocysts decreased when either the folate cycle was inhibited by methotrexate (an antifolate) or BHMT expression was knocked down by antisense morpholinos [79]. Inhibiting both pathways had even more severe impacts on ICM development and cell numbers [79]. Additionally, total SAM levels in mouse blastocysts decreased by almost half and DNA methylation decreased by at least 45%–55% when both BHMT and the folate cycle were inhibited simultaneously [79].

However, betaine supplementation of maternal diets can be detrimental to their offspring if supplementation occurs throughout gestation. Neonatal piglets born to betaine-supplemented sows had higher serum and hepatic betaine contents, higher serum methionine, and greater expression of methionine metabolic enzymes in the liver [80, 81]. Ultimately, this leads to inhibition of hepatic cell proliferation and down-regulation of hepatic expression of the regulatory genes, cyclin D2 and presenilin1 in neonatal piglets [82].

Maternal betaine supplementation increases hepatic cholesterol content in neonatal piglets, through epigenetic regulation of cholesterol metabolic genes [81]. A study conducted in chicks by Hu et al*.* [83] showed that in ovo betaine injections increased serum concentration and hepatic content of cholesterol, through the cholesterol biosynthetic enzyme, 3-hydroxy-3-methylglutaryl-CoA reductase (HMGCR). Additionally, the hepatic protein and mRNA levels of the enzyme that converts cholesterol into bile acids, cholesterol-7 alpha-hydroxylase (CYP7A1), was down-regulated. Furthermore, hepatic protein levels of molecules involved in cholesterol biosynthesis and counter transport increased in betaine treated chicks. The epigenetic effects of betaine were confirmed with alterations in protein content of enzymes involved in the methylation of global genomic DNA and histone H3 in the livers of newly hatched betaine treated chicks [83]. Hu et al*.* [83] reported increased hepatic DNA methyltransferases 1 and adenosylhomocysteinase-like, enzymes associated with genomic DNA hypermethylation, and decreased hepatic histone H3 lysine 27 trimethylation. This histone H3 was recently confirmed as an epigenetic repressor in porcine embryos [84].

It could be inferred that the benefits of feeding high fibre diets prior to mating could be partly through the effects of increased betaine on the developing oocyte.

## Conclusions

Judicial addition of sources of dietary fibre, which are frequently low cost by-products of the food and drink industry, to commercial pig diets provides opportunities to improve several aspects of pig production. These include enhanced welfare through increased satiety and reduced stereotypic behaviours, a reduced environmental footprint and improved reproductive efficiency. Studies described in this review have shown that feeding a diet high in fibre during the period preceding mating is particularly effective. It is believed that this is because the high fibre diets create endocrine profiles and ovarian follicular fluid content that enhance oocyte quality. Such oocytes are then more likely to form viable embryos which, potentially through epigenetic mechanisms, are better able to survive throughout gestation and as piglets. These benefits clearly need to be weighed in the light of the financial implications of dietary alterations, together with the disadvantages of some dietary fibre sources. For example, with respect to feeding USBP during lactation, the hydroscopic characteristics of sugar beet pulp result in low dry matter faeces, with consequent hygiene concerns in farrowing pens and appetite suppression.

For these reasons, studies aimed to (a) identify the most effective dietary fibre, (b) to define the optimal duration and timing of the period when feeding a diet high in fibre would be most beneficial and (c) gain understanding of the underlying mechanisms, in order to identify specific systems or pathways that are modified will be of considerable value to the pig production industry.

## Abbreviations

ATP: Adenosine triphosphate
BHMT: Betaine-homocysteine methyltransferase
CSB: Corn-soybean
CYP7A1: Cholesterol-7 α-hydroxylase
DMSO_2_: Methylsulfonylmethane
DNA: Deoxyribonucleaic acid
E_2_: Oestradiol-17β
FSH: Follicle stimulating hormone
GnRH: Gonadotrophin releasing hormone
HMGCR: 3-hydroxy-3-methylglutaryl-CoA reductase
HP: Hypothalamic-pituitary
ICM: Inner cell mass
IGF-1: Insulin-like growth factor-1
IVF: In vitro fertilisation
IVM: In vitro maturation
LH: Luteinising hormone
M: Maintenance
MII: Metaphase II
NMR: Nuclear magnetic resonance
NPY: Neuropeptide Y
SAM: S-adenosylmethionine
SBH: Soybean hulls
SIT1: Signalling threshold-regulating transmembrane adapter 1
USBP: Unmolassed sugar beet pulp
